# Usefulness of primary care electronic networks to assess the incidence of chlamydia, diagnosed by general practitioners

**DOI:** 10.1186/1471-2296-12-72

**Published:** 2011-07-08

**Authors:** Anita WM Suijkerbuijk, Ingrid VF van den Broek, Henk J Brouwer, Ann M Vanrolleghem, Johanna HK Joosten, Robert A Verheij, Marianne AB van der Sande, Mirjam EE Kretzschmar

**Affiliations:** 1 Centre for Infectious Disease Control, RIVM National Institute of Public Health and the Environment, Bilthoven, The Netherlands; 2 Department of General Practice, Academic Medical Center-University of Amsterdam, The Netherlands; 3 Department of Medical Informatics, Erasmus University Medical Centre, Rotterdam, The Netherlands; 4 Academic General Practice Network VUmc, Free University Medical Centre, Amsterdam, The Netherlands; 5 NIVEL, Netherlands Institute for Health Services Research, Utrecht, The Netherlands; 6 Julius Centre for Health Sciences and Primary Care, University Medical Centre Utrecht, Utrecht, The Netherlands

## Abstract

**Background:**

Chlamydia is the most common curable sexually transmitted infection (STI) in the Netherlands. The majority of chlamydia diagnoses are made by general practitioners (GPs). Baseline data from primary care will facilitate the future evaluation of the ongoing large population-based screening in the Netherlands. The aim of this study was to assess the usefulness of electronic medical records for monitoring the incidence of chlamydia cases diagnosed in primary care in the Netherlands.

**Methods:**

In the electronic records of two regional and two national networks, we identified chlamydia diagnoses by means of ICPC codes (International Classification of Primary Care), laboratory results in free text and the prescription of antibiotics. The year of study was 2007 for the two regional networks and one national network, for the other national network the year of study was 2005. We calculated the incidence of diagnosed chlamydia cases per sex, age group and degree of urbanization.

**Results:**

A large diversity was observed in the way chlamydia episodes were coded in the four different GP networks and how easily information concerning chlamydia diagnoses could be extracted. The overall incidence ranged from 103.2/100,000 to 590.2/100,000. Differences were partly related to differences between patient populations. Nevertheless, we observed similar trends in the incidence of chlamydia diagnoses in all networks and findings were in line with earlier reports.

**Conclusions:**

Electronic patient records, originally intended for individual patient care in general practice, can be an additional source of data for monitoring chlamydia incidence in primary care and can be of use in assessing the future impact of population-based chlamydia screening programs. To increase the usefulness of data we recommend more efforts to standardize registration by (specific) ICPC code and laboratory results across the existing GP networks.

## Background

Genital *Chlamydia trachomatis *infection is the most common curable sexually transmitted infection (STI) in the Netherlands [1]. In men, the most common clinical manifestation of a chlamydia infection is urethritis. In women, it can cause cervicitis and serious complications including upper genital tract infections and it is considered one of the main causes of pelvic inflammatory disease (PID) [2]. Infections are asymptomatic in more than 50% of infected men and 70% of infected women [2]. Since PID and tubal infertility have a large impact on women's reproductive health and treatment is costly, screening programmes have been introduced to control chlamydia through early detection and treatment of asymptomatic infections [3]. Moreover, screening and timely treatment may reduce transmission in the population.

In England, the General Practice Research Database (GPRD) has been used to study incidence of chlamydia diagnoses in primary care [4,5]. In the context of the rollout of the opportunistic National Chlamydia Screening Programme (NSCP), the GRPD proved to be an important source of valuable baseline data. In the Netherlands, the decision to implement population-based screening on a national level is still pending. After a pilot study that assessed the feasibility of screening and the prevalence of chlamydia [6], a large scale Chlamydia Screening Implementation (CSI) project targeting both men and women started in three regions in the Netherlands in 2008 [7,8]. The patient population visiting general practitioners (GPs) and testing positive for chlamydia infection is expected to change in regions where screening is implemented with a substantial coverage. We want to be able to monitor any changes of the incidence of chlamydia diagnoses in primary care. In addition, baseline information of the incidence of chlamydia cases, diagnosed in primary care will be needed to parameterize mathematical models to study the population impact of chlamydia screening programmes [9].

In the Netherlands, the national surveillance of chlamydia is mainly based on data from STI centres, reported to the National Institute of Public Health and the Environment [10]. Dutch STI-centres provide free and anonymous STI testing and care to high-risk groups: persons that fit certain criteria (young age, men who have sex with men, ethnic origin from HIV-endemic areas, and STI-related symptoms). Although surveillance of high-risk groups is important for STIs, the majority of genital chlamydia infections is seen by GPs [7,11,12]. In the Netherlands all inhabitants are registered with a GP; the GP acts as a gatekeeper for secondary care. Reports on the incidence rates of chlamydia diagnoses in primary care are limited. A recent study comparing data from the STI-centre surveillance and electronic medical records of a nationwide sentinel GP network concluded that GPs accounted for 80-85% of STI-diagnoses [13]. The chlamydia reporting rate, based on these two data sources was 220/100,000 population or 36,000 cases reported per year. However, uncertainties remained on the case definition of chlamydia in the GP electronic records.

To obtain more insight into occurrence and reporting of chlamydia diagnoses in GP practice, we compared the incidence of chlamydia diagnoses in the GP network studied in ref 13 with another nationwide and two regional networks. We described the variation in the recording of chlamydia infections and we discussed possible explanations for this variations and the usefulness of these data for the purpose of monitoring chlamydia incidence in the population. This study, together with data form STI-centres, served as baseline information on incidence of chlamydia diagnoses in primary care for the evaluation of the CSI project.

## Methods

### GP networks

For research and education purposes, departments of family medicine of most Dutch universities have primary care networks consisting of GPs from different practices in the region. There are 11 GP networks in the Netherlands which do not overlap. The networks are independent, there is no routine dataflow to a national database. These GP networks collect computer based information about patient care using uniform data collection and registration methods. At regular intervals the information from local registration systems is fed into a central database. On the national level, two GP networks exist that collect data on morbidity, prescriptions and referrals. The aim of regional and national networks is to collect data about primary care in a standardized way, suitable for scientific evaluation. For our study we extracted information about chlamydia diagnoses and treatment from four electronic networks. We selected two nationwide networks as to be able to compare earlier findings in [13] with results form another national network. Since the CSI project was conducted in Amsterdam, Rotterdam and the southern part of Limburg we selected also the two GP networks in Amsterdam. In Rotterdam there was not a regional GP network available; the database of the GP network in Limburg has a focus on a limited number of diagnoses, largely chronic diseases. Therefore, only two regional networks from Amsterdam were included. Ethical approval for this study was not necessary since all patient information in the databases is anonymous, no intervention was done and all patients were informed that their GP participates in scientific research. The four networks: Amsterdam Medical Centre (AMC) network, Academic General Practice Network VU University medical centre (VUmc), Integrated Primary Care Information (IPCI) network and Landelijk Informatie Netwerk Huisartsen (LINH, Netherlands Information Network of General Practice) provided approval for the use of the GP data for this specific study.

### Definition of *Chlamydia trachomatis *diagnoses using ICPC codes

To define chlamydia diagnoses in GP networks, we used a selection of codes within the International Classification of Primary Care (ICPC-1), a worldwide system for coding patient symptoms and diagnoses in primary care [14]. Depending on clinical features chlamydia infections in men and women are recorded under general main-codes that include a broader range of diagnoses (for vaginitis, cervicitis, PID and epididymitis) or specific sub-codes (diagnoses with chlamydia). Since some GPs only register main-codes (ending with .00) and no sub-codes, these main-codes were included only if the treatment (prescribed within two months after the code date) fitted a chlamydia diagnosis. The following antibiotics were included: Azythromycin, Doxycyclin, Amoxicillin, Erythromycin, Ciprofloxacin, and Ofloxacin; they were coded using the Anatomical Therapeutic Chemical (ATC) classification scheme applied by the WHO. We did not use doses of antibiotics.

### Electronic networks and data extraction

#### Amsterdam Medical Centre (AMC) network

Five primary healthcare centres in the south-east of Amsterdam participate in the continuous morbidity registration network of the Department of General Practice, AMC, University of Amsterdam. For our study, we selected all patient records in the year 2007 with a chlamydia-related ICPC code or with the string 'chlam' in the free text. We verified these probable chlamydia diagnoses, based on information of laboratory results in the free text and the prescription of chlamydia-specific antibiotics. Patients' sex, age, date of consultation and duration of registration were used as well as the total number of patients and their time registered in the participating practices in 2007 (patient years).

#### Academic General Practice Network VU University medical center (VUmc)

The VUmc general practitioner network covers 21 practices in the cities Haarlem (13), Amstelveen (1) and Amsterdam (7). Similar to the data extraction of the AMC database, a researcher examined all medical records of 2007 containing the string 'chlam' or a related ICPC code to determine chlamydia episodes. The same patients' characteristics as in the AMC network were available with exception of the time period that patients had been registered in practice.

#### Integrated Primary Care Information (IPCI) network

The IPCI database is a longitudinal observational database which contains data from electronic medical records of 81 practices throughout the Netherlands. Medical records of 2007 were not available; therefore we used all medical records of 2005 to identify chlamydia cases. Like in the networks stated above, records were searched for the string 'chlam' or specific ICPC codes. The identified records were manually validated using all information available in the database to determine the presence of a chlamydia diagnosis. The IPCI database contains demographic information of the patient (date of birth, sex, degree of urbanization of home address, duration of registration), medical notes per consultation (ICPC codes, symptoms, physical examination, assessments and diagnoses), prescriptions, referrals, hospitalizations and laboratory results.

#### Landelijk Informatie Netwerk Huisartsen (LINH) network

LINH is based on electronic medical records from 81 general practices, spread throughout the Netherlands. Data include longitudinal information on patient's characteristics such as age, sex, degree of urbanization of home address, duration of registration in practice, as well as medical information on consultations, prescriptions, referrals, and diagnoses. Based on ICPC codes and ATC codes probable chlamydia infections in 2007 were identified (similar to previous study) [13]. More than in the three other networks, GPs participating in the LINH network are instructed to use ICPC codes for every patient contact. Free text from medical records is not available in this database.

### Data analysis

Chlamydia infections were counted as 'episodes': one or more patient consultations for the same medical diagnosis. In all four networks we included a second episode for the same patient only after an interval of at least two months after the first diagnosis. We assessed diagnoses by ICPC codes, free text and ATC codes in the different networks and calculated incidence of chlamydia diagnoses per age group. The incidence of chlamydia diagnoses was defined as the number of new chlamydia episodes divided by the number of patient years in participating practices in the AMC, IPCI and LINH database. For the denominators of the VUmc network we used the numbers of patients at the end of the year. We also described the incidence of diagnosed cases by level of population density in the LINH and IPCI network.

In accordance with Statistics Netherlands the following definitions for population density were used:

• Level 1: highly urban: 2,500 addresses or more per square km

• Level 2: 1,500 to 2,500 addresses per square km

• Level 3: 1,000 to 1,500 addresses per square km

• Level 4: 500 to 1,000 addresses per square km

• Level 5: rural: fewer than 500 addresses per square km

## Results

### Characteristics of patient populations in the four networks

Table 1 shows a number of characteristics of the study population of the selected networks and the way chlamydia was defined. The size of study population in the different networks varied from 35,137 to 327,725 depending on the number of practices included. In all networks slightly more women than men were registered. The patients in the catchment area of the AMC network had a lower socioeconomic status and were more often of non-western origin than in the other networks. The AMC and VUmc network both covered a highly urbanized population, whereas the two national networks contain practices from areas with a population density representative for the whole country.

**Table 1:** Overview of general practice networks

	AMC network	VUmc network	IPCI	LINH
Region	South-east Amsterdam	Amsterdam, Haarlem, Amstelveen	Nationwide	Nationwide

Year of data extraction	2007	2007	2005	2007

No of practices	5	21	81	81

Size of network population	35,137	66,402	235,307	327,725

Percentage of women	53%	53%	51%	51%

Patient characteristics	Lower socioeconomic status and more often non-western descent than the general population; living in an highly urbanized area	More often of Dutch descent; living in an highly urbanized area	Representative sample of the general population [21]	Representative sample of the general population [22]

Definition of chlamydia	A chlamydia diagnosis was based on laboratory results, appropriate antibiotics or correct ICPC code in the medical record	Idem, based on laboratory results, antibiotics and ICPC code	Idem, based on laboratory results, antibiotics and ICPC code	A chlamydia diagnosis was based on a specific ICPC sub-code or main-code combined with a chlamydia-related antibiotic

Overall incidence rate per 100,000 (95% CI)	590.2 (506.7 - 687.3)	275.4 (233.6 - 324.7)	103.2 (89.2 - 118.7)	195.9 (179.3 - 214.1)

### Use of ICPC codes

The use of ICPC codes in the identified (probable) chlamydia episodes in the electronic records varied largely among the four networks (table 2) for both men and women. The proportion of episodes identified with any ICPC code differed. More than 40% of the chlamydia diagnoses in the AMC and IPCI network did not have an ICPC code, while in the VUmc network more than 90% and in the LINH network all records contained a code. The availability of ICPC codes facilitated the search for chlamydia diagnoses in VUmc and LINH networks. Second, there were differences in the use of the various ICPC codes in the GP networks. In the VUmc and LINH network the code for PID (X74 and X74.01) was found more frequently than in the AMC and IPCI network. In all networks except the VUmc network code X84.00 was sometimes used, while this is not an appropriate code to define a chlamydia diagnosis. Unlike the AMC and LINH networks, a considerable part of the probable chlamydia cases in the VUmc and IPCI network were registered under the code X99.00 (other genital disease in women). In the LINH network, the main-codes (X74.00, X84.00, and Y99.00) and sub-code Y74.02 (epididymitis) were seen more frequently than in the other networks. Finally, the proportion of episodes defined by sub-codes specific for chlamydia varied largely, this was 40.6% of chlamydia cases in the IPCI-network 48.2% of chlamydia diagnoses at AMC practices, 68% in the LINH-network and 74.5% in the VUmc-network.

**Table 2:** Use of ICPC code in different GP networks for patients identified in our study as having a chlamydia diagnosis

Reported ICPC codes	Explanation code	AMC 2007% (N = 164, 49 men; 115 women)	VUmc 2007% (N = 141, 55 men; 86 women)	IPCI 2005% (N = 189, 80 men; 109 women)	LINH 2007% (N = 485, 229 men; 256 women)
No code	Verified in free text and in laboratory results	43	7.1	42.9	0

X74.00	PID	2.4	2.1	2.6	10.5
X74.04					

X74.01	PID by chlamydia	-	8.5	-	2.7

X74.05	Other pelvic infection	-	-	2.1	-

X84.00	Vaginitis	1.8	-	2.1	10.5

X84.01	Vaginitis by chlamydia	17.7	10.6	-	12.2

X85.00	Cervicitis	0.6	-	5.3	3.3

X85.01*	Cervicitis by chlamydia	15.9	19.9	5.3	13

X85.04	Other cervical disease	-	-	0.5	-

X99.00	Genital disease or sexually transmitted infection in women	1.2	16.3	14.8	-
X99.03					
X99.05					

Y74.00	Orchitis/Epididymitis	0.6	-	1.1	16.7
Y74.01					
Y74.02					

Y99.00	Genital disease or sexually transmitted infection in men	1.8	-	23.3	7.8
Y99.01					
Y99.05					
Y99.06					

Y99.03	Genital disease by chlamydia in men	14	35.5	-	22.9

Y99.07	Unknown code	-	-	-	0.4

Total		100	100	100	100

*In the IPCI network: cervicitis

### Incidence of chlamydia diagnoses in the GP networks

**Figure 1:**
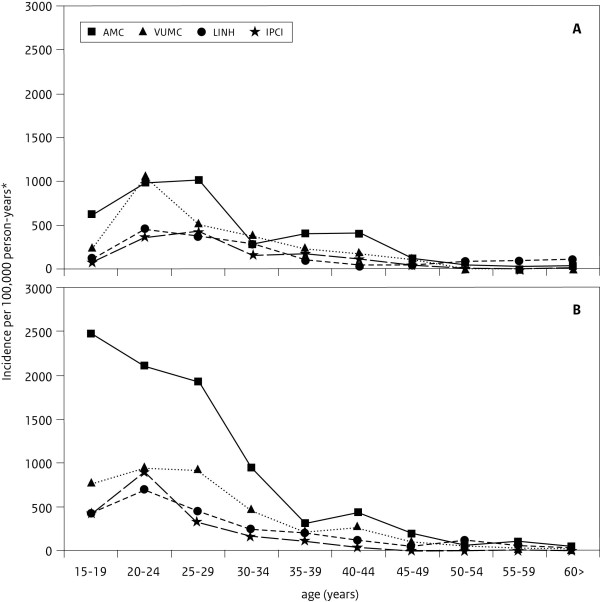
**Incidence of chlamydia diagnoses per 100,000 person-years in men (A) and women (B) in primary care networks**. (* in the VUmc network, total number of patients)

The estimated incidence of chlamydia diagnoses was higher in the regional networks around Amsterdam than in the nationwide networks. The incidence rate was 590.2/100,000 for AMC, and 275.4/100,000 for VUmc compared to 103.2/100,000 for IPCI and 195.9/100,000 for LINH network (table 1). The incidence rates were lower in men than in women and peaked in different age groups (see Figure 1a and 1b). In the LINH and VUmc networks incidence rates were highest for men in the age group 20-24 years old (461 and 1060 per 100,000 respectively) while in the AMC and IPCI network men from 25 to 29 years had the highest incidence of chlamydia diagnoses (1012 and 423 per 100,000 respectively). With increasing age numbers of chlamydia diagnoses declined in all the networks. In the LINH network, men aged 50 years and older had a relatively higher incidence rate than in the other networks. Highest rates in women were seen in the AMC network and especially in the youngest age group of 15 to 19 years old (2466 per 100,000). The VUmc, LINH and IPCI network had the highest incidence in the age group 20 to 24 years old (936, 702 and 906 per 100,000 respectively).

### Incidence of chlamydia diagnoses related to population density

For the nationwide networks IPCI and LINH, we further stratified the incidence rates by degree of urbanization (table 3). The highest rates were seen in the highly urbanized areas in men and women at the age of 15 to 29. In rural areas a few groups showed incidence rates higher than in semi-rural areas. The overall incidence of chlamydia diagnoses in the youngest age groups was similar in both networks: in men 297 and 325 per 100,000, in women 535 and 525 in IPCI and LINH network respectively.

**Table 3:** Incidence of chlamydia diagnoses per 100,000 person-years in IPCI and LINH network concerning degree of urbanization

Degree of urbanization	Men all ages		Women all ages		men 15-29		women 15-29	
	IPCI	LINH	IPCI	LINH	IPCI	LINH	IPCI	LINH

Level 1: highly urban	132.5	340.8	156.2	371.7	427.4	634.1	650.3	881.1
95%CI	93-184	277-419	112-212	307-450	276-635	471-853	453-906	703-1105

Level 2	96.6	197.7	144.4	248.5	341.5	364.2	616.1	682.1
95%CI	61-146	141-278	99-203	185-333	196-558	217-611	405-901	472.-984

Level 3	54.9	181.2	67.8	166.3	140.7	255.4	330.8	331.9
95%CI	33-87	130-253	43-103	118-235	63-276	243-622	197-524	201-547

Level 4	52.1	138.9	146.7	97.1	201.8	210.3	686.8	261.5
95%CI	22-107	103-187	89-230	68-139	68-480	125.-353	385-1141	163-418

Level 5: rural	75.6	108.3	101.8	173.6	282.3	83.1	498.1	441.0
95%CI	21-202	73-161	34-242	126-239	56-905	32-214	138-1329	286-680

Overall	87.3	189.7	119.1	207.9	296.3	325.3	535.9	525.1
95%CI	70-108	167-216	98.-143	184-235	225-389	265-399	435-660	450-613

## Discussion

Our study showed that electronic databases from primary care networks can be used to estimate the incidence of chlamydia diagnoses. However, we observed variability in the incidence rate reported by the four networks; different registration rules/habits and socio-demographic characteristics of the patient populations in the networks contributed to this variability. Diagnosed cases of chlamydia infection were recorded in several ways in GP electronic records. While most GPs used ICPC codes for recording details of their diagnosis, others resorted to free text fields from which extracting standardized diagnoses is difficult and time-consuming. The application of ICPC codes in general as well as the use of specific (sub)codes differed from network to network hampering comparability and the analysis of overall trends.

The estimated incidence of chlamydia diagnoses varied from 103 to 590 per 100,000 in the four networks. Despite the large difference in incidence rates, similar trends were observed. Chlamydia incidence was highest in the youngest age groups (15 to 30 years), where incidence rates in women were approximately twice as high as in men. This is in agreement with what is found in Dutch STI clinics, where the numbers of young women diagnosed with chlamydia are higher than in young men [1]. The highest incidence rates of diagnosed cases were found in the AMC network. Patients included in the AMC network, in the south-eastern part of Amsterdam have on average a lower socioeconomic status and are more often of non-western origin. Based on a classification of Statistics Netherlands and The Netherlands Institute for Social Research 92% of all patients in these practices belonged to the lowest of five socioeconomic status levels while 53% were of non-western descent, in 2007. Other studies revealed that the risk of acquiring chlamydia is associated with ethnicity and social economic status [6,15]. In addition, the southeast of Amsterdam is a highly urbanized area and as in the IPCI and LINH network we observed a general increase in chlamydia incidences with higher degree of urbanization. In contrast, the relatively high incidence in rural areas in the two national networks is remarkable. Although it cannot be excluded that this reflects a chance effect due to low numbers, further investigations into testing practices are needed to explore this finding.

Our results regarding the trends in incidence of chlamydia diagnoses by age, sex and degree of urbanization compare well with a large population-based chlamydia screening pilot study conducted in 2002/2003. In that study, the prevalence of chlamydia was found to be 2% among 15-29 years old participants (1.5% in men and 2.5% in women). Prevalence was lower in rural areas (0.6%) compared to very highly urbanized areas (3.2%) [6]. Assuming an (untreated) chlamydia infection lasts on average one year, these prevalence rates can be roughly interpreted as annual incidence rates [16-18], which would mean an incidence rate of 1500 and 2500 per 100,000 in young men and women. Our findings in the nationwide networks (an incidence of 300 and 500 per 100,000 in young men and women) indicate that about 20% of the total incident chlamydia cases in that age group is diagnosed by GPs.

Because chlamydia was not recorded under one code and reporting laboratory results was not standard, uncertainty remains about whether all of the extracted cases were actually chlamydia cases and about how many cases of chlamydia were recorded in a way that they were not identified by our extraction rules. The definition of a chlamydia infection that uses fixed main-codes in combination with the code for a specific antibiotic treatment in the LINH network could potentially lead to an overestimation of chlamydia incidence especially in higher age groups. For example, the antibiotic treatment for epididymitis in older men is also prescribed for other pathogens than *Chlamydia trachomatis*. Information about chlamydia testing results were lacking for the majority of patients in these networks. Finally, the years of study were not the same for every network, which could have influenced the observed incidence rates.

A study of morbidity rates in general practice registration networks in the Netherlands identified potential reasons for the observed variability that apply to our findings as well [19]. We also perceived differences in the methodological characteristics of the networks, such as definitions and registration rules used. Another reason for variability pertains to the variety of testing practices used among GPs. A qualitative study exploring the reasons for variation in diagnostic testing found major differences between high and low chlamydia testers [20]. Possibly the rate of testing is higher in an urban setting compared to a rural area, explaining at least partly the difference in incidence rates in both areas. The last reason these authors mentioned was related to the socio-demographic characteristics of the catchment population of the networks, which is certainly true for the differences between the national and regional networks in our study, as described above.

## Conclusion

In this study, we have demonstrated that electronic medical records, originally intended for individual patient care in general practice, can be an additional source of information about incidence of chlamydia as diagnosed in primary care. These estimates would be helpful to assess the future impact of population-based chlamydia screening programs. In view of the considerable variation in the way chlamydia has been coded and reported, we recommend that more efforts should be undertaken to standardize registration rules and record-taking for GP networks. In meetings of representatives of the GP networks, methods of registration could be discussed and standardized. The implementation of a single ICPC code for chlamydia infections, as it is defined in ICPC-2 classification, preferably combined with recording of laboratory results in all GP practices that are connected to a data-collecting network is necessary to achieve better comparability of GP registries.

## Competing interests

The authors declare that they have no competing interests.

## Authors' contributions

AS was involved in analysing and interpreting the data and drafted the manuscript. IB initiated and designed the study and revised the manuscript critically. HB, AV, JJ and RV coordinate a GP network and its data acquisition, advised and commented on earlier versions of the manuscript. MS and MK revised and corrected the draft. All authors read and approved the final manuscript.

## Pre-publication history

The pre-publication history for this paper can be accessed here:

http://www.biomedcentral.com/1471-2296/12/72/prepub
